# Distinct Brain Functional Impairment Patterns Between Suspected Non-Alzheimer Disease Pathophysiology and Alzheimer’s Disease: A Study Combining Static and Dynamic Functional Magnetic Resonance Imaging

**DOI:** 10.3389/fnagi.2020.550664

**Published:** 2020-11-23

**Authors:** Zheyu Li, Kaicheng Li, Xiao Luo, Qingze Zeng, Shuai Zhao, Baorong Zhang, Minming Zhang, Yanxing Chen, for the Alzheimer’s Disease Neuroimaging Initiative

**Affiliations:** ^1^Department of Neurology, the Second Affiliated Hospital, School of Medicine, Zhejiang University, Hangzhou, China; ^2^Department of Radiology, the Second Affiliated Hospital, School of Medicine, Zhejiang University, Hangzhou, China

**Keywords:** suspected non-Alzheimer disease pathophysiology, Alzheimer’s disease, amplitude of low-frequency fluctuation, dynamic brain activity, resting-state fMRI

## Abstract

**Background**: Suspected non-Alzheimer disease pathophysiology (SNAP) refers to the subjects who feature negative β-amyloid (Aβ) but positive tau or neurodegeneration biomarkers. It accounts for a quarter of the elderly population and is associated with cognitive decline. However, the underlying pathophysiology is still unclear.

**Methods**: We included 111 non-demented subjects, then classified them into three groups using cerebrospinal fluid (CSF) Aβ 1–42 (A), phosphorylated tau 181 (T), and total tau (N). Specifically, we identified the normal control (NC; subjects with normal biomarkers, A-T-N-), SNAP (subjects with normal amyloid but abnormal tau, A−T+), and predementia Alzheimer’s disease (AD; subjects with abnormal amyloid and tau, A+T+). Then, we used the static amplitude of low-frequency fluctuation (sALFF) and dynamic ALFF (dALFF) variance to reflect the intrinsic functional network strength and stability, respectively. Further, we performed a correlation analysis to explore the possible relationship between intrinsic brain activity changes and cognition.

**Results**: SNAP showed decreased sALFF in left superior frontal gyrus (SFG) while increased sALFF in left insula as compared to NC. Regarding the dynamic metric, SNAP showed a similarly decreased dALFF in the left SFG and left paracentral lobule as compared to NC. By contrast, when compared to NC, predementia AD showed decreased sALFF in left inferior parietal gyrus (IPG) and right precuneus, while increased sALFF in the left insula, with more widely distributed decreased dALFF variance across the frontal, parietal and occipital lobe. When directly compared to SNAP, predementia AD showed decreased sALFF in left middle occipital gyrus and IPG, while showing decreased dALFF variance in the left temporal pole. Further correlation analysis showed that increased sALFF in the insula had a negative correlation with the general cognition in the SNAP group. Besides, sALFF and dALFF variance in the right precuneus negatively correlated with attention in the predementia AD group.

**Conclusion**: SNAP and predementia AD show distinct functional impairment patterns. Specifically, SNAP has functional impairments that are confined to the frontal region, which is usually spared in early-stage AD, while predementia AD exhibits widely distributed functional damage involving the frontal, parietal and occipital cortex.

## Introduction

Suspected non-Alzheimer disease pathophysiology (SNAP) refers to the subjects with abnormal tau or neurodegeneration but normal amyloid deposition (Jack et al., 2018). Previous studies have usually focused on Alzheimer’s continuum and ignored these subjects due to the lack of Alzheimer’s disease (AD) core biomarker β-amyloid (Aβ). However, recent epidemiological investigations claimed that SNAP accounts for about a quarter in cognitively normal (CN) and mild cognitive impairment (MCI) population (Jack et al., 2012, 2016; Schreiber et al., 2017). Furthermore, longitudinal studies found a greater cognitive decline in SNAP patients than in subjects with normal biomarkers (Caroli et al., 2015; Vos et al., 2015; Chung et al., 2017; Jack et al., 2017; Ben Bouallègue et al., 2018). Thus, understanding the underlying pathophysiology of SNAP is necessary.

Previous studies reported that tau deposition in SNAP is located in the bilateral medial and lateral temporal region (Dodich et al., 2020), as well as hypometabolism in temporoparietal regions (Schreiber et al., 2017; Chiaravalloti et al., 2019). This is somehow in line with the magnetic resonance imaging (MRI) findings, which found more severe baseline hippocampal atrophy in SNAP than normal control (NC; Caroli et al., 2015; Burnham et al., 2016; Gordon et al., 2016; Chung et al., 2017). Though the above study gave us some insight into the foundation of the cognitive decline of SNAP, the functional impairment pattern of SNAP is still unknown.

Amplitude of low-frequency fluctuation (ALFF) is an effective neuroimaging index in reflecting neurodegenerative changes caused by different pathologies. To be specific, the static ALFF (sALFF) reflects the regional intrinsic functional activity strength by calculating the average ALFF signal through the whole resting-state period (Yang et al., 2007). Decreased sALFF associates with impaired brain activity, while increased sALFF is usually regarded as a compensatory mechanism to cognitive impairment in neurodegenerative diseases (Palacios et al., 2013; Liu et al., 2016; Yang et al., 2018). On the other hand, the dynamic ALFF (dALFF) could reflect the temporal variability of intrinsic brain activity (Fu et al., 2018). Abnormalities in dynamic brain activity, including excessive variability (increased dALFF) and excessive stability (decreased dALFF), impair brain function (Christoff et al., 2016). These methods have been widely used in neurodegenerative diseases, like AD (Yang et al., 2018; de Vos et al., 2018; Li et al., 2019; Zeng et al., 2019), Parkinson’s disease (Skidmore et al., 2013; Zhang et al., 2019) have been proven feasible and effective. Thus, combining the sALFF and dALFF may help to illuminate the underlying brain functional changes in SNAP.

In our study, we aim to explore the brain functional changes in SNAP by combining the sALFF and dALFF. Notably, to compare the differences in functional impairment patterns, we included the predementia AD group (A+T+) as a reference. According to previous findings (Caroli et al., 2015; Jack et al., 2016; Altomare et al., 2019; Lowe et al., 2019) that SNAP had a relatively distinct pathological background and clinical trajectory from AD, we hypothesized that SNAP might display different functional changes relative to AD.

## Materials and Methods

### Alzheimer’s Disease Neuroimaging and Initiative

All data used in this article were downloaded from the Alzheimer’s Disease Neuroimaging Initiative (ADNI) database^1^. The ADNI is a longitudinal multicenter study since 2004 and now contains ADNI 1, ADNI GO, ADNI 2, and ADNI 3. By the use of clinical, neuropsychological assessment, gene, biofluid, and imaging data, it aims to investigate the biomarkers of early detection and progression of AD.

### Study Participants

All individuals in this study signed the written informed consent as they joined the ADNI project. We identified 111 non-demented subjects (characterized as either CN or MCI) from the ADNI GO/2database (the flowchart was presented in Supplementary Material 1; Table 1). We included the CN and MCI since SNAP is much more prevalent in the non-demented population than in the dementia population (Dani et al., 2017; Yu et al., 2019). All these subjects had undergone structural MRI, resting-state functional MRI (rsfMRI), lumbar puncture, and comprehensive neuropsychological assessments.

**Table 1:** Demographic and clinical characteristics.

	NC (A-T-N-)	SNAP (A-T+)	Predementia AD (A+T+)	*F*(*χ*^2^)	*P*-value
**Demographics**
Number	17	29	65	−	−
Female	13 (76.47%)	13 (44.83%)	31 (47.69%)	5.14	0.08
Age (years)	71.89 ± 6.03	73.92 ± 8.60	74.10 ± 6.67	0.66	0.52
APOEε4	2 (11.76%)	6 (20.69%)	40 (61.54%)	21.74	<0.001
Education (years)	15.88 ± 3.28	16.55 ± 2.43	16.25 ± 2.46	0.37	0.70
MCI	10 (58.82%)	15 (51.72%)	40 (61.54%)	0.39	0.68
GDS	1.24 ± 1.60	0.93 ± 0.92	1.14 ± 1.17	0.44	0.65
**General mental status**
MMSE	28.35 ± 1.54	28.59 ± 1.50	27.82 ± 2.07	1.90	0.15
CDR global	0.26 ± 0.26	0.26 ± 0.25	0.35 ± 0.30	1.44	0.24
**Memory**
WMS-LM immediate	12.00 ± 4.34	12.00 ± 3.28	10.58 ± 4.61	1.34	0.27
WMS-LM delayed	10.19 ± 4.20	10.28 ± 4.31	8.37 ± 4.77	2.02	0.14
AVLT sum of trials 1–5	41.00 ± 11.96	42.24 ± 10.65	36.68 ± 10.75	3.02	0.05
AVLT recognition	11.65 ± 3.06	12.17 ± 2.70	11.31 ± 3.01	0.86	0.43
**Visuo-spatial function**
CDT	4.71 ± 0.59	4.48 ± 0.74	4.51 ± 0.85	0.50	0.61
**Language**
BNT	27.82 ± 2.04	28.31 ± 1.69	27.43 ± 3.52	0.90	0.41
Category Fluency Test	18.88 ± 4.85	21.52 ± 5.88	19.49 ± 5.22	1.82	0.17
**Attention**
Log-transformed TMT-A	3.55 ± 0.28	3.47 ± 0.33	3.56 ± 0.30	1.02	0.36
**Executive function**
Log-transformed TMT-B	4.38 ± 0.33	4.38 ± 0.43	4.50 ± 0.45	1.00	0.37
**CSF Biomarkers**
Aβ_1–42_ (pg/ml)	229.00 ± 31.96^c^	244.52 ± 29.01^c^	141.26 ± 23.96^ab^	182.31	<0.001
P-tau_181_ (pg/ml)	17.48 ± 3.93^bc^	34.55 ± 9.92^ac^	53.28 ± 24.52^ab^	26.06	<0.001
T-tau (pg/ml)	47.24 ± 15.87^c^	64.72 ± 23.32^c^	102.67 ± 49.77^ab^	16.84	<0.001

*Data are presented as mean (SD) or number (%). Abbreviations: SD, standard deviation; NC, normal control; SNAP, suspected non-Alzheimer’s pathophysiology; AD, Alzheimer’s disease; GDS, Geriatric Depression Scale; MMSE, Mini-Mental State Examination; CDR: Clinical Dementia Rating; WMS-LM, Wechsler Memory Scale Logical Memory; AVLT, Auditory Verbal Learning Test; CDT, Clock Drawing Test; BNT, Boston Naming Test; TMT, Trail-Making Test. ^a^Significantly different compared to A-T-N-; ^b^significantly different compared to A−T+; ^c^significantly different compared to A+T+*.

Cognitively normal was defined as: (1) Clinical Dementia Rating (CDR) = 0; (2) Mini-Mental State Exam (MMSE) score between 24 and 30 (inclusive); (3) Normal Wechsler Memory Scale Logical Memory (WMS-LM) delay recall performance (in detail: ≥9 for subjects with 16 or more years of education; ≥5 for subjects with 8–15 years of education; and ≥3 for 0–7 years of education); (4) Without memory complaints; (5) No impairment in cognitive functions or activities of daily living. MCI was defined as (1) CDR = 0.5; (2) MMSE score between 24 and 30 (inclusive); (3) Abnormal WMS-LM delay recall performance documented by scoring within the education adjusted ranges; (4) Subjective memory concern reported by the subject itself, study partners or clinician; and (5) Preserved general cognition and functional performance so that a diagnosis of AD dementia cannot be made.

We excluded individuals with following manifestations: (1) Significant medical, neurologic, and psychiatric illness, such as Parkinson’s disease, major depression, clinically significant abnormalities in vitamin B12; (2) Obvious head trauma history; (3) Use of non-AD related medication known to influence cerebral function; and (4) Alcohol or drug abuse (more details about the inclusion and exclusion criteria were presented in Supplementary Material 2).

### Neuropsychological Assessments

All subjects completed the comprehensive cognitive assessment (Table 1), including general mental status assessed by MMSE and CDR global, memory assessed by Auditory Verbal Learning Test (AVLT) and WMS-LM, attention assessed by Trail-Making Test Part A (TMT-A), executive function assessed by Trail-Making Test Part B (TMT-B), visuospatial function assessed by Clock-Drawing Test (CDT), and language assessed by Boston Naming Test (BNT) and Category Fluency Test.

### Group Classifications

Cerebrospinal fluid (CSF) β-amyloid 1–42 (Aβ1–42), phosphorylated tau at position 181 (P-tau181), and total tau (T-tau) are core AD biomarkers (Jack et al., 2018). The non-dementia stages enable early prevention and intervention. Thus, we classified people based on the CSF biomarkers of AD to explore the imaging characteristics in the non-dementia stage.

According to the 2018 National Institute on Aging-Alzheimer’s Association (NIA-AA) research framework (Jack et al., 2018), we use CSF Aβ_1–42_, T-tau, and P-tau_181_ level as the classification criteria. The CSF samples were measured by the multiplex xMAP Luminex platform as previously described (Olsson et al., 2005; Shaw et al., 2009). We set the CSF cutoff point at 192 pg/ml for Aβ_1–42_, 23 pg/ml for P-tau_181_, and 93 pg/ml for T-tau (Shaw et al., 2009). Decreased CSF Aβ_1–42_ and elevated CSF P-tau_181_or T-tau levels were regarded as abnormalities. Then, we divided 111 non-demented subjects into three groups: NC: subjects with normal Aβ_1–42_ and P-tau_181_ and T-tau (A-T-N-, *n* = 17), SNAP: subjects with normal Aβ_1–42_ and abnormal P-tau_181_ (A-T+, *n* = 29), predementia AD: subjects with abnormal Aβ_1–42_ and P-tau_181_ (A+T+, *n* = 65). Due to the limited number of A+T-, we did not include this group for analysis (the flowchart in Supplementary Material 1).

### MRI Acquisition

The T1 structure images were obtained using Three-dimensional Magnetization Prepared Rapid Acquisition Gradient Echo (3D MPRAGE) T1-weighted sequence with the following parameters: voxel size = 1.0 × 1.0 × 1.2 mm^3^; flip angle = 9°; echo time (TE) = 3.13 ms; repetition time (TR) = 6.77 ms; 170 sagittal slices; within plane FOV = 256 × 256 mm^2^. The rsfMRI images were obtained using an echo-planar imaging sequence with the following parameters: 140 time points; TE = 30 ms; TR = 3,000 ms; number of slices = 48; slice thickness = 3.3 mm; spatial resolution = 3.31 × 3.31 × 3.31 mm^3^; flip angle = 80°; matrix = 64 × 64. All subjects undergo MRI scanning with their eyes open, focusing on a cross, and kept at rest calmly according to the ADNI scanning protocol.

### MRI Pre-processing

The rsfMRI data were preprocessed using the Data Processing Assistant for Resting-state fMRI (DPARSF^2^, Chao-Gan and Yu-Feng, 2010) based on the platform of Statistical Parametric Mapping 12 (SPM12)^3^. First, the first 10 volumes of rsfMRI scans were removed due to the signal equilibrium and the subject’s adaptation to the scanning noise. The remaining 130 images were corrected for both timing differences between each slice and head motion (Friston 24-parameter model (Friston et al., 1996). Next, image data with head motion displacement of not more than 2.5 mm in any of the *x*, *y*, or *z* directions or 2.5° rotation of angular motion were chosen for further analysis (one SNAP and one predementia AD subject was excluded). Then, T1-weighted images and the mean rsfMRI images were co-registered, spatially normalized to the Montreal Neurological Institute (MNI) standard space, and subsequently re-sampled into 3 mm × 3 mm × 3 mm cubic voxel. Nuisance covariates, including 24 head motion parameters and signals of white matter and CSF, were corrected. Finally, rsfMRI images were detrended and spatially smoothed with a Gaussian kernel of 6 × 6 × 6 mm^3^ full width at half maximum (FWHM).

### sALFF and dALFF Variance Calculation

The sALFF was computed using the DPARSF toolbox to reflect the strength of intrinsic brain activity. The procedure was as follows: the time series of each voxel was changed into the frequency domain with a fast Fourier transform. Next, across the 0.01–0.08 Hz domain, the square root of the power spectrum in each voxel was computed and averaged. This averaged square root was taken as the sALFF of each voxel (Zang et al., 2007). Finally, to standardize the result, the sALFF of each voxel was divided by the global mean sALFF value within the default brain mask from the DPARSF.

The dALFF was computed in the DynamicBC toolbox^4^ (Liao et al., 2014) by using a sliding window approach to reflect the dynamic change of intrinsic brain activity. According to previous studies which proved that window sizes in the range of 40 s to 100 s could capture brain dynamics well (Zalesky and Breakspear, 2015), we chose 14TR (42 s) as the window size, and 1TR as the window step. Then, we got an ALFF map for each sliding window, as well as the dALFF variance that reflects the temporal stability of intrinsic brain activity. To test the reliability of the results under different window sizes, we also analyzed dALFF in other window sizes, and details were presented in Supplementary Material 3.

### Statistical Analysis

Demographic data were analyzed in SPSS (version 23.0) by using Chi-squared (*χ*^2^) test for categorical data (gender, APOE4 genotyping) and analysis of variance (ANOVA) for continuous data (age, education years, neuropsychological scores, and CSF biomarkers). Then, *post hoc* analysis using two-sample *t*-tests was further performed to reveal the source of ANOVA difference (*P* < 0.05, corrected by Bonferroni).

We adopted a voxel-wise two-sample *t*-test in the DPABI toolbox (Yan et al., 2016) to explore the neuroimaging metric differences (including sALFF, dALFF variance) between SNAP and NC, predementia AD and NC, as well as predementia AD and SNAP, with gray matter volume and age as covariates. We set the threshold at 0.01 for voxel *P*-value, 0.05 for cluster *P*-value using the Gaussian random field (GRF) correction.

We defined regions with significantly changed sALFF and dALFF variance as the regions of interest (ROIs) and extracted each subjects’ values of sALFF and dALFF within ROIs. Then, we performed partial correlation analyses between the sALFF or dALFF of ROIs and neurophysiological or pathological data after controlling for age. In detail, pathological data included CSF Aβ_1–42_, T-tau, and P-tau_181_. Neurophysiological data included general mental status (MMSE, CDR global), memory (AVLT, WMS-LM), attention (TMT-A), executive function (TMT-B), visuospatial function (CDT), and language (BNT, Category Fluency Test). To reduce the effects of multiple comparisons, we chose *P* < 0.01 as the statistical significance level.

## Results

### Demographics, Cognitive and CSF Data

We presented the demographics, CSF biomarkers levels, and neuropsychological scores in Table 1. There is no significant statistical difference in age, gender, education, clinical diagnosis, and geriatric depression scale (GDS) among three groups (*P* > 0.05). The proportion of APOEε4 carriers was significantly lower in SNAP (20.7%) and NC (11.8%) than that in the predementia AD group (61.5%, *P* < 0.001).

As for cognitive data, three groups did not differ significantly in general mental status (MMSE, CDR global) and multiple cognitive domains (memory, visuospatial function, language, attention, and executive function; *P* > 0.05).

As for the CSF pathological biomarkers, P-tau_181_ showed significant changes in SNAP and predementia AD group (*P* < 0.001). Aβ_1–42_ and T-tau showed significant changes only in the predementia AD group (*P* < 0.001).

### sALFF Result

**Figure 1:**
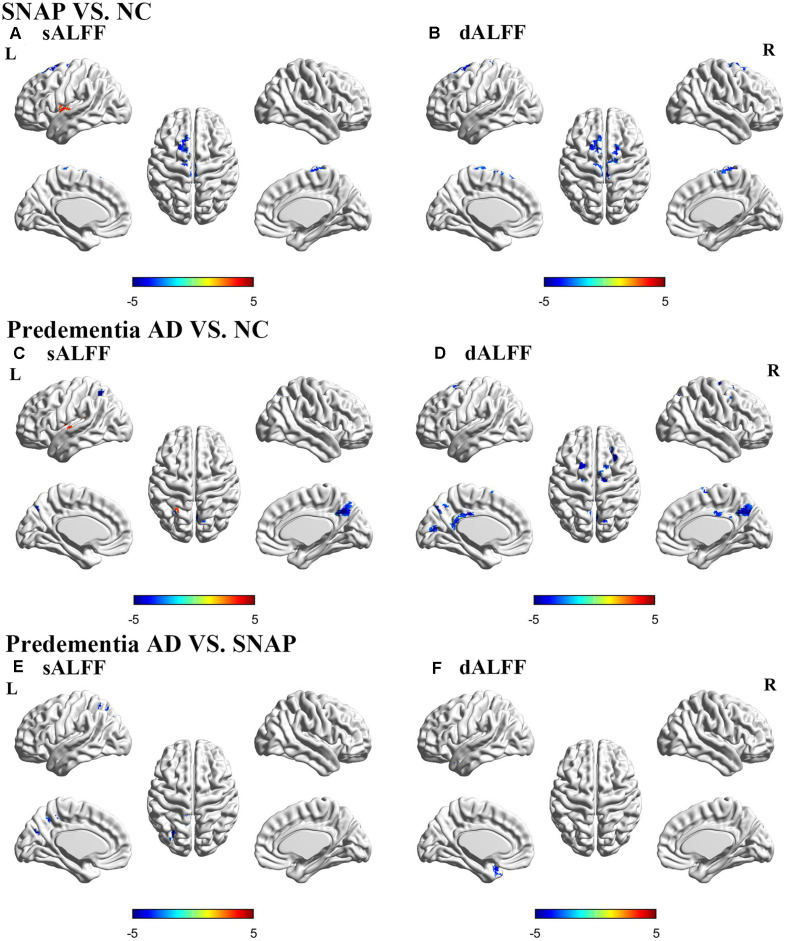
Brain areas with significant differences of sALFF and dALFF in SNAP and predementia AD. Upper panel: SNAP vs. NC; Middle panel: predementia AD vs. NC; Lower panel: predementia AD vs. SNAP. **(A,C,E)** Differences of sALFF; **(B,D,F)** differences of dALFF [voxel *P* < 0.01, cluster *P* < 0.05, controlling for age, gray matter volume, Gaussian random field (GRF) corrected]. Abbreviations: sALFF, static amplitude of low-frequency fluctuation; dALFF, dynamic amplitude of low-frequency fluctuation; NC, normal control; SNAP, suspected non-Alzheimer’s pathophysiology; AD, Alzheimer’s disease.

SNAP had decreased sALFF in the left superior frontal gyrus (SFG), and increased sALFF in the left insula when compared to NC (Figure 1A, Table 2, Supplementary Material 4; voxel *P* < 0.01, cluster *P* < 0.05, controlling age and gray matter volume, GRF corrected). Predementia AD showed decreased sALFF in the right precuneus, left inferior parietal gyrus (IPG) while increased sALFF in the left insula than NC (Figure 1C, Table 2, Supplementary Material 4). When directly compared to SNAP, predementia AD showed decreased sALFF in left middle occipital gyrus and left IPG (Figure 1E, Table 2, Supplementary Material 4).

**Table 2:** Brain areas with significant differences of sALFF and dALFF in SNAP and predementia AD.

Neuroimaging metrics	Group	Regions	Peak MNI			Cluster size	Peak intensity
			*x*	*y*	*z*		
sALFF	SNAP vs. NC	Left paracentral lobule	−12	−24	72	149	−4.67
		Left rolandic operculum	−42	−9	18	77	4.06
	Predementia AD vs. NC	Right precuneus	15	−69	48	203	−5.55
		Left inferior parietal gyrus	−24	−54	51	73	−7.67
		Left Heschl’s gyrus	−57	−9	9	88	4.23
	Predementia AD vs. SNAP	Left middle occpital gyrus	−30	−69	30	89	−5.26
		Left inferior parietal gyrus	−24	−54	54	105	−5.92
dALFF variance	SNAP vs. NC	Left superior frontal gyrus	−18	12	63	85	−4.53
		Left paracentral lobule	−12	−24	72	125	−5.04
	Predementia AD vs. NC	Left calcarine	−6	−78	12	68	−4.93
		Left middle cingulum	−3	−27	33	87	−4.40
		Right middle frontal gyrus	27	33	33	74	−4.94
		Right precuneus	18	−69	48	159	−5.23
		Right supplementary motor area	15	−12	66	81	−5.07
		Left superior frontal gyrus	−18	9	63	82	−5.27
	Predementia AD vs. SNAP	Left temporal pole: superior temporal gyrus	−36	12	−27	78	−4.60

*Statistical significance was set at voxel *P* < 0.01, cluster *P* < 0.05, controlling for age, gray matter volume, Gaussian random field (GRF) corrected. Abbreviations: MNI, Montreal Neurological Institute; sALFF, static amplitude of low-frequency fluctuation; dALFF, dynamic amplitude of low-frequency fluctuation; NC, normal control; SNAP, suspected non-Alzheimer’s pathophysiology; AD, Alzheimer’s disease. More details about the regions were put in Supplementary Material 4*.

### dALFF Variance Result

SNAP showed decreased dALFF variance in left SFG and left paracentral lobule when compared to NC (Figure 1B, Table 2, Supplementary Material 4; voxel *P* < 0.01, cluster *P* < 0.05, controlling age and gray matter volume, GRF corrected), while predementia AD group showed decreased dALFF variance in a wide cortical area involving left calcarine, left middle cingulum, right precuneus, right supplementary motor area, left SFG, and right middle frontal gyrus (MFG) when compared to NC (Figure 1D, Table 2, Supplementary Material 4). Moreover, as directly compared with SNAP, predementia AD showed decreased dALFF variance in the left temporal pole (Figure 1F, Table 2, Supplementary Material 4).

### Correlation Analysis

**Figure 2:**
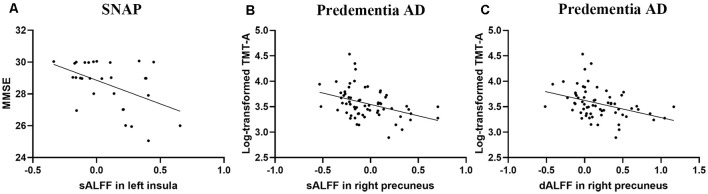
Scatter plot diagram of the correlation between sALFF/dALFF and cognition. **(A)** Increased sALFF in insula correlated with worse MMSE in SNAP group (*r* = −0.485, *P* = 0.009, corrected for age). **(B,C)** The sALFF/dALFF in precuneus negatively correlated with Log-transformed TMT-A finish time in predementia AD group (*r* = −0.390, *P* = 0.001; *r* = −0.375, *P* = 0.002; corrected for age, respectively). Abbreviations: sALFF, static amplitude of low-frequency fluctuation; dALFF, dynamic amplitude of low-frequency fluctuation; SNAP, suspected non-Alzheimer’s pathophysiology; AD, Alzheimer’s disease; MMSE, Mini-Mental State Examination; TMT-A, Trail-Making Test Part-A.

We performed partial correlation analyses between the functional changes and cognition, as well as pathological biomarkers to explore the physiological significance of the sALFF and dALFF (detailed results were presented in Supplementary Material 5). For SNAP, we found that sALFF in the insula had a negative correlation with MMSE (*r* = −0.485, *P* = 0.009, corrected for age, Figure 2A). As for predementia AD, sALFF and dALFF in precuneus were associated with attention (Log-transformed TMT-A, *r* = −0.390, *P* = 0.001; *r* = −0.375, *P* = 0.002; corrected for age, respectively, Figures 2B,C). We did not find a significant correlation between CSF biomarkers and functional changes in SNAP and predementia AD.

## Discussion

To the best of our knowledge, this is the first study investigating the brain functional changes in SNAP by combining the sALFF and dALFF. Our study found distinct impairment patterns in SNAP and predementia AD as compared to NC: SNAP showed decreased intrinsic functional connectivity strength and stability in SFG, while also showing increased intrinsic functional connectivity strength in the insula, while predementia AD showed widespread decreased functional connectivity strength and stability involving the frontal, parietal and occipital cortex. In the direct comparison of SNAP and predementia AD group, predementia AD showed decreased functional connectivity strength and stability than SNAP. Accordingly, SNAP is a different neurodegenerative disease entity from AD, which needs more clinical attention.

### Distinct Brain Functional Impairment Patterns in SNAP and Predementia AD

Our findings on predementia AD showed decreased sALFF in the right precuneus and left IPG, while increased sALFF in the left insula. These results are largely consistent with the classic network disconnectivity theory in AD: reduced functional connectivity in default mode network (DMN), especially the precuneus and IPG, while salience network (SN) can be activated as compensation to maintain cognitive integrity (Zhou et al., 2010). Thereinto, precuneus, as the vital component of DMN, is vulnerable to suffer amyloid deposition and functional changes at the early AD stage and is significantly correlated with cognitive changes (Palmqvist et al., 2017). This can also be proved by our study which observed the significant correlation between intrinsic brain activity within precuneus and attention in predementia AD. Moreover, our dALFF results extensively showed widespread decreased intrinsic brain activity stability in predementia AD subjects, which suggested progressively widespread functional network disruption involving DMN, SN, and executive control network (ECN) in AD (Menon, 2011; Zhao et al., 2019).

On the other hand, SNAP showed a quite different impaired pattern: decreased sALFF and dALFF variance in SFG while increased sALFF in the insula. First, the altered functional signal in SFG suggested the decreased intrinsic brain activity strength and stability in SFG. The decreased brain activity strength and stability in SFG represented the specific impairment pattern of SNAP, which differs from the compensatory function of SFG in AD patients (Maillet and Rajah, 2013; Franzmeier et al., 2018). Functionally, SFG works as the core structure of the ECN and contributes to high cognitive functions including working memory and executive function (du Boisgueheneuc et al., 2006; Alagapan et al., 2019; Briggs et al., 2020). A recent study showed that SNAP (A−T+) had worse frontal lobe function performance than AD (A+T+) in the dementia population (Takenoshita et al., 2019). Accordingly, the decreased brain functional connectivity strength and flexibility in SFG may be the key to cognition changes in SNAP.

Moreover, we also found increased sALFF in the insula, suggesting the increased intrinsic brain activity strength. Functionally, The insula plays a crucial role in SN and mediates the interaction between large-scale functional networks during cognitive processes (Menon and Uddin, 2010; Chen et al., 2016). The insula involves multiple functions, including sensory, emotional, autonomic, and cognitive function (Menon and Uddin, 2010; Gasquoine, 2014). Previous studies found increased functional connectivity in the SN in subjects with neurodegenerative diseases and regarded it as a compensatory response to decreased cognitive ability (Yassa et al., 2008; Agosta et al., 2012; Skouras et al., 2019). Similarly, increased cerebral blood flow in the insula has also been identified as a compensatory mechanism against pathological damage in the preclinical phase of AD (Caroli et al., 2010; Fazlollahi et al., 2020). Our further analysis also observed a positive association between sALFF and MMSE in SNAP, supporting the compensatory mechanism. Conclusively, SNAP showed functional impairments in SFG with the compensatory improvement of function in the insula. This is partially distinct from the classic AD impairment pattern.

When directly compared to SNAP, predementia AD showed significantly decreased sALFF in left middle occipital gyrus and left IPG, and decreased dALFF variance in the left temporal pole. This result indicated that AD had more functional impairment than SNAP in the non-dementia stage, which further supports our finding that SNAP has distinct brain functional impairment patterns from predementia AD.

### The Possible Mechanism Underlying SNAP

SNAP features functional changes in the SFG and insula. This is quite different from the classic AD functional change pattern: widespread functional impairments starting from DMN while SFG is spared at the early stage. The possible mechanism may be the distinct pathological deposit and distribution. SNAP features abnormal Tau and normal Amyloid, while AD here features both abnormal Amyloid and Tau. Moreover, another study found that entorhinal tauopathy correlated with distant frontal hypometabolism in an Aβ independent way (Hanseeuw et al., 2017), which might be the possible reason for frontal functional damage in SNAP. Medial temporal lobe tauopathy without amyloid was common in the brain of older people, which was thought to account for a subset of SNAP population (Crary et al., 2014). A recent tau position emission tomography (PET) study supported this finding and showed bilateral temporal lobe tau deposition in SNAP (Dodich et al., 2020). As for AD, amyloid deposition spreads widely in the neocortex early in the pathological processes (Braak and Braak, 1991), which may explain why AD showed widespread cortical functional changes. Moreover, the synergistic effect of amyloid deposition and pathologic tau may induce more impairments than amyloid deposition or tau alone (Bloom, 2014; He et al., 2018).

There are some limitations in our study. First, the sample size is relatively small, which may reduce statistical power and would not allow for a subgroup analysis between different cognitive stages of SNAP. Further studies with larger sample sizes should be performed. Second, SNAP is a relatively heterogeneous group, easily resulting from other factors like cerebrovascular disease (CVD; Wong et al., 2019), primary age-related tauopathy (PART; Vos et al., 2013) or argyrophilic grain disease (AGD; Lowe et al., 2019). Further studies should consider these associated factors. Third, as for dALFF, there lacks a unified criterion for the window size in the sliding-window analysis process. We explored the analysis in other window sizes to prove the stability of the result. Moreover, a longer scan length for the dALFF might be better to study the pattern of dynamic brain activity. At last, we did not find significant differences in the cognitive scores between SNAP and NC (Table 1). This might be explained by the relatively intact cognition of the included subjects, and that pathological changes and fMRI abnormalities always precede cognitive changes (Sperling et al., 2011; Sheline and Raichle, 2013). Further studies with longitudinal data may give more hints on the clinical implications of SNAP.

## Conclusion

SNAP shows impaired functional activities mainly confined to the frontal lobe and activated insula, while predementia AD shows widespread functional activity changes involving frontal, parietal, and occipital cortex as compared to NC. Moreover, such decreased intrinsic brain activity strength and stability in SNAP may explain its cognitive decline and rapid clinical progression. Our study suggests that SNAP can be distinguished from AD both pathologically and functionally, and more attention should be put on SNAP.

## Data Availability Statement

The datasets generated and/or analyzed during the current study are available in the ADNI study. More details in www.adni-info.org.

## Ethics Statement

All procedures performed in studies involving human participants were in accordance with the ethical standards of the institutional and/or national research committee and with the 1964 Helsinki declaration and its later amendments or comparable ethical standards.

Written informed consent was obtained from all participants and/or authorized representatives and the study partners before any protocol-specific procedures were carried out in the ADNI study.

## Author Contributions

ZL collected and analyzed the MRI data, and wrote the first draft of the manuscript. KL analyzed the MRI data and wrote the protocol. YC and MZ designed and conceptualized the study, and revised the manuscript. ZL, QZ, SZ, and BZ assisted with study design and interpretation of findings. All authors contributed to the article and approved the submitted version.

## Conflict of Interest

The authors declare that the research was conducted in the absence of any commercial or financial relationships that could be construed as a potential conflict of interest.

## Footnotes

^1^http://adni.loni.usc.edu/

^2^http://rfmri.org/DPARSF

^3^www.fil.ion.ucl.ac.uk/spm

^4^www.restfmri.net/forum/DynamicBC
