# Supplementary material for: Distinct Brain Functional Impairment Patterns Between Suspected Non-Alzheimer Disease Pathophysiology and Alzheimer’s Disease: A Study Combining Static and Dynamic Functional Magnetic Resonance Imaging
Source: Front Aging Neurosci. 2020 Nov 23;12:550664. doi: 10.3389/fnagi.2020.550664 (PMC7719833; doi:10.3389/fnagi.2020.550664)
Supplement: Supplementary file 1 [file supplementary_material.DOC]

**Distinct brain functional impairment patterns between suspected non-Alzheimer disease pathophysiology and Alzheimer’s disease: a study combining static and dynamic functional MRI**

Zheyu Li^1#^, Kaicheng Li^2#^, Xiao Luo^2^, Qingze Zeng^2^, Shuai Zhao^1^, Baorong Zhang^1^, Minming Zhang^2^*, Yanxing Chen^1^* for the Alzheimer’s Disease Neuroimaging Initiative^‡^

^1^Department of Neurology, the Second Affiliated Hospital, School of Medicine, Zhejiang University, Hangzhou, China

^2^Department of Radiology, the Second Affiliated Hospital, School of Medicine, Zhejiang University, Hangzhou, China

Corresponding to:

*Prof. Yanxing Chen, MD, PhD; Department of Neurology, the Second Affiliated Hospital, School of Medicine, Zhejiang University, No.88 Jiefang Road, Shangcheng District, Hangzhou, China, 310009; Email address: [chenyanxing@zju.edu.cn](mailto:chenyanxing@zju.edu.cn)

*Prof. Minming Zhang, MD, PhD; Department of Radiology, the Second Affiliated Hospital, School of Medicine, Zhejiang University, No.88 Jiefang Road, Shangcheng District, Hangzhou, China, 310009; Phone: 86-0571-87315255; Fax: 86-0571-87315255; Email address: [zhangminming@zju.edu.cn](mailto:zhangminming@zju.edu.cn)

^‡^Data used in the preparation of this article were obtained from the Alzheimer’s Disease Neuroimaging Initiative (ADNI) database (adni.loni.usc.edu). As such, the investigators within the ADNI contributed to the design and implementation of ADNI and/or provided data but did not participate in analysis or writing of this report. A complete listing of ADNI investigators can be found at: <http://adni.loni.usc.edu/wp-content/uploads/how_to_apply/ADNI_Acknowledgement_List.pdf>

^#^Zheyu Li and Kaicheng Li were contributed equally to this work

**Supplementary Material 1. Flow chart of subject inclusion**

We firstly identified 136 cognitively normal (CN) or mild cognitive impairment (MCI) subjects who has a 3T structural magnetic resonance imaging (MRI) scan, resting-state functional MRI (rsfMRI) scan, and cerebrospinal fluid (CSF) sample from Alzheimer's Disease Neuroimaging Initiative (ADNI) GO/2 database. After carefully screening the images, 3 subjects were excluded due to abnormal geriatric depression scale (GDS) score. 15 subjects were excluded due to different rsfMRI parameters or bad image quality, and 1 subject was excluded due to cerebral infarction lesions, and 2 subjects were excluded due to abnormal head motion. Then, 115 subjects were classified basing on CSF amyloid-β 1-42 (Aβ_1-42_, A), phosphorylated tau 181 (P-tau_181_, T), and total tau (T-tau, T) level (Shaw et al., 2009). Given that there were only 4 people in the A+T- group, this group was not included in our analysis. We finally included 111 subjects from three groups: normal control (NC): subjects with normal Aβ_1-42_, P-tau_181_, and T-tau (A-T-N-, n=17); suspected non-Alzheimer disease pathophysiology (SNAP): subjects with normal Aβ_1-42_ and abnormal P-tau_181_ (A-T+, n=29); predementia Alzheimer’s disease (AD): subjects with abnormal Aβ_1-42_ and P-tau_181_ (A+T+, n=65). The flow chart was presented as follows (eFigure 1).

**eFigure 1** Flow chart of subject inclusion


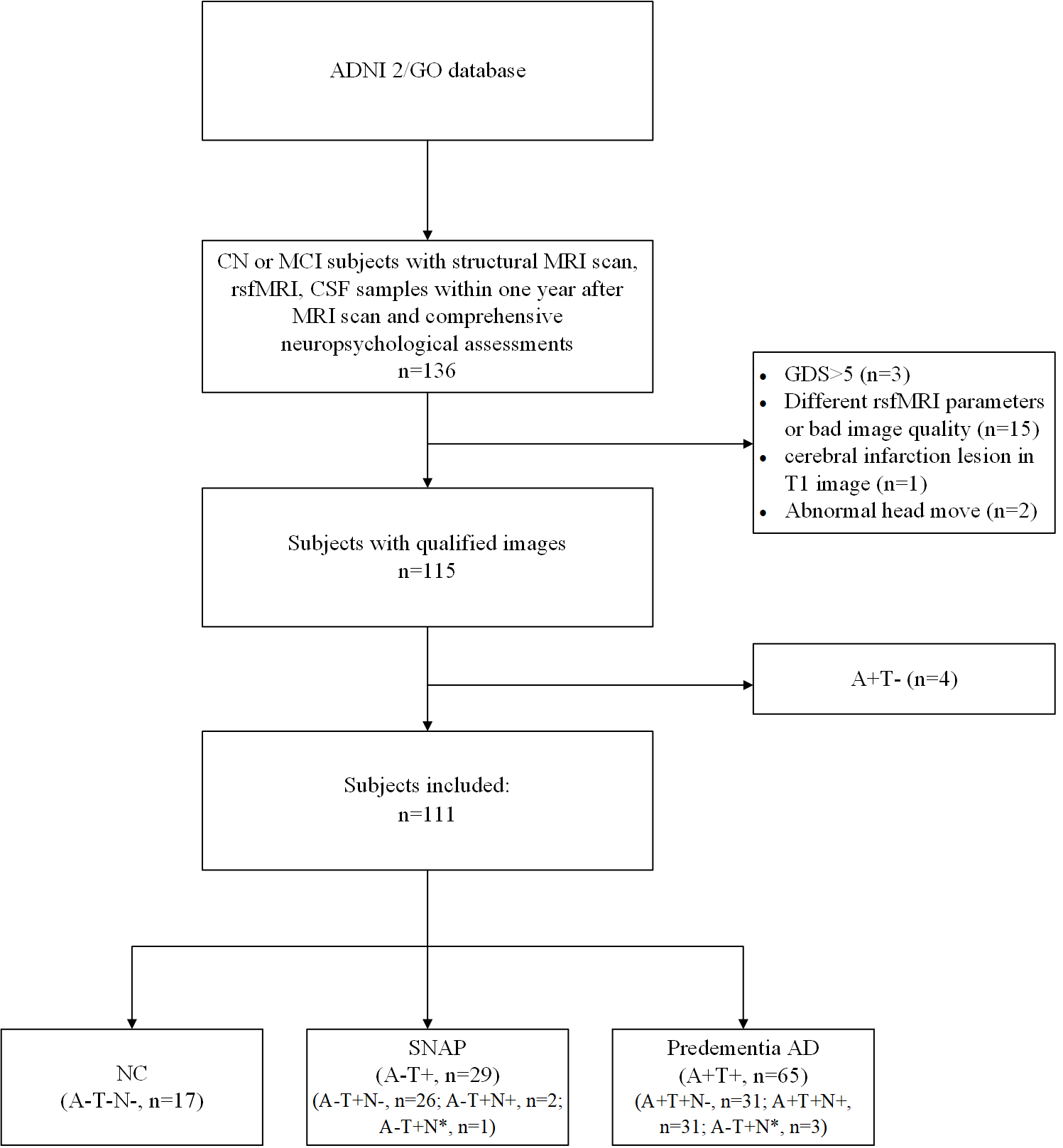


**Abbreviation:** ADNI, Alzheimer’s Disease Neuroimaging Initiative; CN, cognitively normal; MCI, mild cognitive impairment; MRI, magnetic resonance imaging; rsfMRI, resting-state functional MRI; CSF, cerebrospinal fluid; GDS, geriatric depression scale; NC, normal control; SNAP, suspected non-Alzheimer’s pathophysiology; AD, Alzheimer’s disease; A-, amyloid normal using CSF Aβ_1-42_; A+, amyloid abnormal using CSF Aβ_1-42_; T-, tau normal using CSF P-tau_181_; T+, tau abnormal using CSF P-tau_181_; N-, neurodegeneration or neuronal injury normal using CSF T-tau; N+, neurodegeneration or neuronal injury abnormal using CSF T-tau; N*, unavailable for CSF T-tau

**Supplementary Material 2. The subject’s** **inclusion and exclusion criteria**

All subjects were from ADNI GO/2. Subjects were included or excluded following the ADNI GO/2 protocol. The full list of inclusion and exclusion criteria could be found in <http://adni.loni.usc.edu/wp-content/themes/freshnews-dev-v2/documents/clinical/ADNI-2_Protocol.pdf> (Page 18-22). The definitions of CN and MCI were described in the main text. Additional inclusion criteria were as follow:

1. Geriatric Depression Scale less than 6.

2. Hachinski less than or equal to 4.

3. Visual and auditory acuity adequate for neuropsychological testing.

4. Age between 55-90 (inclusive).

5. Completed six grades of education or has a good work history (sufficient to exclude mental retardation).

6. Good general health with no diseases expected to interfere with the study.

7. The subject is not pregnant, lactating, or of childbearing potential (i.e. women must be two years post-menopausal or surgically sterile).

8. Must speak English or Spanish fluently.

9. No medical contraindications to magnetic resonance imaging (MRI).

10. Willing, able to and agree to participate in the study.

Individuals with the following manifestations were excluded:

1. Any significant neurologic disease, such as Parkinson’s disease, multi-infarct dementia, Huntington’s disease, normal pressure hydrocephalus, brain tumor, progressive supranuclear palsy, seizure disorder, subdural hematoma, multiple sclerosis, or history of significant head trauma followed by persistent neurologic defaults or known structural brain abnormalities.

2. Any significant psychiatric illness, such as a history of schizophrenia (DSM IV criteria), major depression or bipolar disorder (DSM-IV) within the past 1 year.

3. Clinically significant abnormalities in B12, or Thyroid Function Tests (TFTs) that might interfere with the study.

4. Use of non-AD related medication known to inﬂuence cerebral function, like certain antidepressants, neuroleptics, chronic anxiolytics, or sedative-hypnotics.

5. History of alcohol or substance abuse or dependence within the past 2 years (DSM IV criteria).

6. Presence of contraindications of MRI, including pacemakers, aneurysm clips, artificial heart valves, ear implants, metal fragments, or foreign objects in the eyes, skin or body.

**Supplementary Material 3. Significant inter-group differences in dALFF analysis with other window sizes**

To test the reliability of our results under different window sizes, we also analyzed dynamic amplitude of low-frequency fluctuation (dALFF) in the window size of 20, 26, 33 repetition time (TR) (eTable 1). The results were similar to that in 14 TR that SNAP showed decreased dALFF in the left superior frontal gyrus (SFG), while predementia AD group showed decreased dALFF involving frontal, parietal, and occipital lobe in a more diffused pattern.

**eTable 1** Significant inter-group differences in dALFF analysis with other window sizes

| **Window sizes** | **Group** | **Anatomical area** | **Peak MNI** | | | **Cluster Size** | **Peak intensity** |
| --- | --- | --- | --- | --- | --- | --- | --- |
|  |  |  | **x** | **y** | **z** |  |  |
| **20 TR** | **SNAP vs. NC** | Left superior frontal gyrus Left supplementary motor area | -18 | 12 | 63 | 78 | -4.61 |
|  | **Predementia AD vs. NC** | Left calcarine | -12 | -81 | 12 | 82 | -5.10 |
|  |  | Left middle cingulum Left post cingulum | -3 | -27 | 33 | 85 | -4.37 |
|  |  | Right middle frontal gyrus | 27 | 33 | 33 | 67 | -4.93 |
|  |  | Right precuneus | 18 | -69 | 48 | 157 | -6.15 |
|  |  | Left superior parietal gyrus | -21 | -54 | 51 | 59 | -6.71 |
|  |  | Right superior frontal gyrus Right supplementary motor area | 12 | -6 | 66 | 78 | -4.60 |
|  |  | Left supplementary motor area Left superior frontal gyrus Left paracentral lobule | -15 | -9 | 63 | 70 | -5.73 |
| **26 TR** | **SNAP vs. NC** | Left superior frontal gyrus Left supplementary motor area | -9 | 21 | 63 | 71 | -4.50 |
|  | **Predementia AD vs. NC** | Left calcarine | -6 | -81 | 12 | 84 | -5.54 |
|  |  | Left middle cingulum | 0 | -27 | 33 | 52 | -4.37 |
|  |  | Right precuneus | 18 | -69 | 48 | 131 | -6.81 |
|  |  | Right inferior parietal gyrus | 30 | -48 | 51 | 65 | -4.68 |
|  |  | Right superior frontal gyrus Right supplementary motor area | 9 | 0 | 69 | 83 | -4.91 |
| **33 TR** | **SNAP vs. NC** | Left superior frontal gyrus Left supplementary motor area | -9 | 9 | 69 | 71 | -4.53 |
|  | **Predementia AD vs. NC** | Left calcarine Left lingual gyrus | -6 | -81 | 12 | 102 | -5.58 |
|  |  | Right precuneus | 18 | -69 | 48 | 138 | -6.26 |
|  |  | Left superior parietal gyrus Left inferior parietal gyrus | -27 | -54 | 54 | 49 | -5.39 |
|  |  | Right superior frontal gyrus Right supplementary motor area | 12 | -6 | 66 | 73 | -4.75 |
| Statistical significance was set at *P*<0.01 at voxel level, *P*<0.05 at the cluster level, controlling for age, grey matter volume, Gaussian random field (GRF) corrected  Abbreviations: dALFF, dynamic amplitude of low-frequency fluctuation; MNI, Montreal Neurological Institute; TR, repetition time; NC, normal control; SNAP, suspected non-Alzheimer’s pathophysiology; AD, Alzheimer’s disease | | | | | | | |

**Supplementary Material 4. Brain areas with significant differences of sALFF and dALFF in SNAP and predementia AD**

**eTable 2** Brain areas with significant differences of sALFF and dALFF in SNAP and predementia AD

| **Neuroimaging Metrics** | **Group** | **Regions** | **Peak MNI** | | | **Cluster Size** | **Peak intensity** |
| --- | --- | --- | --- | --- | --- | --- | --- |
|  |  |  | **x** | **y** | **z** |  |  |
| **sALFF** | **SNAP vs. NC** | Left superior frontal gyrus, Left supplementary motor area, Left paracentral lobule | -12 | -24 | 72 | 149 | -4.67 |
|  |  | Left insula, Left Rolandic operculum, Left superior temporal gyrus | -42 | -9 | 18 | 77 | 4.06 |
|  | **Predementia AD vs. NC** | Right precuneus | 15 | -69 | 48 | 203 | -5.55 |
|  |  | Left superior parietal gyrus, Left inferior parietal gyrus | -24 | -54 | 51 | 73 | -7.67 |
|  |  | Left insula, Left Rolandic operculum, Left superior temporal gyrus, Left Heschl's gyrus | -57 | -9 | 9 | 88 | 4.23 |
|  | **Predementia AD vs. SNAP** | Left middle occpital gyrus, Left superior occpital gyrus | -30 | -69 | 30 | 89 | -5.26 |
|  |  | Left superior parietal gyrus, Left precuneus, Left inferior parietal gyrus | -24 | -54 | 54 | 105 | -5.92 |
| **dALFF variance** | **SNAP vs. NC** | Left superior frontal gyrus, Left supplementary motor area | -18 | 12 | 63 | 85 | -4.53 |
|  |  | Left paracentral lobule | -12 | -24 | 72 | 125 | -5.04 |
|  | **Predementia AD vs. NC** | Left calcarine | -6 | -78 | 12 | 68 | -4.93 |
|  |  | Left middle cingulum, Left post cingulum | -3 | -27 | 33 | 87 | -4.40 |
|  |  | Right middle frontal gyrus | 27 | 33 | 33 | 74 | -4.94 |
|  |  | Right precuneus | 18 | -69 | 48 | 159 | -5.23 |
|  |  | Right superior frontal gyrus, Right supplementary motor area | 15 | -12 | 66 | 81 | -5.07 |
|  |  | Left superior frontal gyrus, Left supplementary motor area, Left paracentral lobule | -18 | 9 | 63 | 82 | -5.27 |
|  | **Predementia AD vs. SNAP** | Left temporal pole: superior temporal gyrus, Left temporal pole: middle temporal gyrus | -36 | 12 | -27 | 78 | -4.60 |

**Supplementary Material 5. The correlation analysis between the functional changes and cognition in SNAP and predementia AD**

**eTable 3 The correlation analysis between the functional changes and cognition in SNAP**

|  | ROIs (sALFF) |  | ROIs (dALFF ) |  |
| --- | --- | --- | --- | --- |
|  | Left insula | Left SFG | Left SFG | Left paracentral lobule |
| **General mental status** |  |  |  |  |
| MMSE | r=-0.485, *P*=0.009* | r=0.401, *P*=0.035 | r=0.344, *P*=0.073 | r=0.254, *P*=0.193 |
| CDR global | r=0.050, *P*=0.800 | r=0.015, *P*=0.942 | r=0.003, *P*=0.987 | r=0.171, *P*=0.385 |
| **Memory** |  |  |  |  |
| WMS-LM immediate | r=0.185, *P*=0.386 | r=-0.258, *P*=0.224 | r=-0.341, *P*=0.103 | r=-0.247, *P*=0.245 |
| WMS-LM delayed | r=0.311, *P*=0.139 | r=-0.185, *P*=0.388 | r=-0.299, *P*=0.156 | r=-0.154, *P*=0.472 |
| AVLT sum of trials 1-5 | r=0.012, *P*=0.950 | r=0.173, *P*=0.380 | r=0.087, *P*=0.659 | r=0.101, *P*=0.611 |
| AVLT recognition | r=-0.079, *P*=0.690 | r=0.263, *P*=0.176 | r=0.241, *P*=0.217 | r=0.257, *P*=0.186 |
| **Visuo-spatial function** |  |  |  |  |
| CDT | r=-0.074, *P*=0.710 | r=-0.041, *P*=0.837 | r=0.147, *P*=0.454 | r=-0.175, *P*=0.373 |
| **Language** |  |  |  |  |
| BNT | r=-0.121, *P*=0.540 | r=0.081, *P*=0.681 | r=0.118, *P*=0.548 | r=-0.042, *P*=0.833 |
| Category Fluency Test | r=0.002, *P*=0.993 | r=0.150, *P*=0.447 | r=0.050, *P*=0.802 | r=0.009, *P*=0.963 |
| **Attention** |  |  |  |  |
| Log-transformed TMT-A | r=0.146, *P*=0.458 | r=-0.143, *P*=0.468 | r=-0.097, *P*=0.623 | r=-0.021, *P*=0.914 |
| **Executive function** |  |  |  |  |
| Log-transformed TMT-B | r=-0.178, *P*=0.366 | r=-0.054, *P*=0.786 | r=-0.128, *P*=0.518 | r=0.109, *P*=0.582 |
| **CSF Biomarkers** |  |  |  |  |
| Aβ_1-42_ (pg/ml) | r=0.007, *P*=0.972 | r=0.015, *P*=0.939 | r=-0.010, *P*=0.960 | r=-0.005, *P*=0.979 |
| P-tau_181_ (pg/ml) | r=0.265, *P*=0.172 | r=-0.374, *P*=0.050 | r=-0.400, *P*=0.035 | r=-0.232, *P*=0.234 |
| T-tau (pg/ml) | r=0.092, *P*=0.649 | r=0.105, *P*=0.603 | r=-0.071, *P*=0.725 | r=0.073, *P*=0.717 |
| Abbreviations: sALFF, static amplitude of low-frequency fluctuation; dALFF, dynamic amplitude of low-frequency fluctuation; SFG, superior frontal gyrus; MMSE, Mini-Mental State Examination; CDR: Clinical Dementia Rating; WMS-LM, Wechsler Memory Scale Logical Memory; AVLT, Auditory Verbal Learning Test; CDT, Clock Drawing Test; BNT, Boston Naming Test; TMT, Trail-Making Test. * Significantly significant (*P*＜0.01) | | | | |

**eTable 4** The correlation analysis between the functional changes in sALFF and cognition for predementia AD

|  | ROIs (sALFF) | | |
| --- | --- | --- | --- |
|  | Left insula | Right precuneus | Left IPG |
| **General mental status** |  |  |  |
| MMSE | r=-0.019, *P*=0.884 | r=-0.146, *P*=0.249 | r=0.013, *P*=0.916 |
| CDR global | r=0.202, *P*=0.110 | r=0.080, *P*=0.527 | r=0.007, *P*=0.957 |
| **Memory** |  |  |  |
| WMS-LM immediate | r=-0.049, *P*=0.713 | r=-0.012, *P*=0.928 | r=0.130, *P*=0.329 |
| WMS-LM delayed | r=-0.103, *P*=0.439 | r=-0.098, *P*=0.463 | r=0.023, *P*=0.863 |
| AVLT sum of trials 1-5 | r=-0.092, *P*=0.469 | r=-0.011, *P*=0.928 | r=-0.088, *P*=0.491 |
| AVLT recognition | r=0.054, *P*=0.672 | r=-0.107, *P*=0.406 | r=-0.146, *P*=0.253 |
| **Visuo-spatial function** |  |  |  |
| CDT | r=0.012, *P*=0.922 | r=0.059, *P*=0.643 | r=0.037, *P*=0.772 |
| **Language** |  |  |  |
| BNT | r=-0.007, *P*=0.958 | r=0.129, *P*=0.310 | r=0.088, *P*=0.488 |
| Category Fluency Test | r=-0.040, *P*=0.756 | r=0.203, *P*=0.108 | r=0.165, *P*=0.193 |
| **Attention** |  |  |  |
| Log-transformed TMT-A | r=0.026, *P*=0.837 | r=-0.389, *P*=0.001* | r=-0.248, *P*=0.048 |
| **Executive function** |  |  |  |
| Log-transformed TMT-B | r=0.214, *P*=0.095 | r=-0.252, *P*=0.049 | r=-0.142, *P*=0.271 |
| **CSF Biomarkers** |  |  |  |
| Aβ_1-42_ (pg/ml) | r=-0.166, *P*=0.190 | r=-0.040, *P*=0.757 | r=0.029, *P*=0.817 |
| P-tau_181_ (pg/ml) | r=0.226, *P*=0.073 | r=-0.160, *P*=0.205 | r=0.016, *P*=0.899 |
| T-tau (pg/ml) | r=0.136, *P*=0.297 | r=-0.199, *P*=0.125 | r=-0.213, *P*=0.099 |
| Abbreviations: sALFF, static amplitude of low-frequency fluctuation; dALFF, dynamic amplitude of low-frequency fluctuation; IFG, inferior parietal gyrus; MMSE, Mini-Mental State Examination; CDR, Clinical Dementia Rating; WMS-LM, Wechsler Memory Scale Logical Memory; AVLT, Auditory Verbal Learning Test; CDT, Clock Drawing Test; BNT, Boston Naming Test; TMT, Trail-Making Test. * Significantly significant (*P*＜0.01) | | | |

**eTable 5**  The correlation analysis between the functional changes in dALFF and cognition for predementia AD

|  | ROIs (dALFF) | | | | | |
| --- | --- | --- | --- | --- | --- | --- |
|  | Left calcarine | Left middle cingulum | Right MFG | Right supplementary motor area | Left SFG | Right precuneus |
| **General mental status** |  |  |  |  |  |  |
| MMSE | r=-0.095, *P*=0.456 | r=0.137, *P*=0.280 | r=0.177, *P*=0.161 | r=0.148, *P*=0.242 | r=-0.068, *P*=0.591 | r=-0.145, *P*=0.252 |
| CDR global | r=0.045, *P*=0.722 | r=-0.105, *P*=0.411 | r=-0.105, *P*=0.409 | r=-0.087, *P*=0.495 | r=0.044, *P*=0.731 | r=0.089, *P*=0.484 |
| **Memory** |  |  |  |  |  |  |
| WMS-LM immediate | r=0.075, *P*=0.576 | r=0.138, *P*=0.301 | r=0.163, *P*=0.223 | r=0.096, *P*=0.473 | r=0.052, *P*=0.696 | r=0.001, *P*=0.995 |
| WMS-LM delayed | r=-0.113, *P*=0.399 | r=0.093, *P*=0.488 | r=0.157, *P*=0.240 | r=0.061, *P*=0.652 | r=-0.030, *P*=0.826 | r=-0.095, *P*=0.477 |
| AVLT sum of trials 1-5 | r=-0.138, *P*=0.278 | r=0.083, *P*=0.514 | r=0.190, *P*=0.132 | r=0.198, *P*=0.118 | r=0.087, *P*=0.494 | r=0.011, *P*=0.930 |
| AVLT recognition | r=-0.138, *P*=0.282 | r=0.123, *P*=0.338 | r=0.023, *P*=0.857 | r=0.109, *P*=0.397 | r=-0.135, *P*=0.293 | r=-0.094, *P*=0.463 |
| **Visuo-spatial function** |  |  |  |  |  |  |
| CDT | r=0.005, *P*=0.966 | r=0.185, *P*=0.144 | r=0.111, *P*=0.382 | r=0.130, *P*=0.304 | r=-0.063, *P*=0.620 | r=0.027, *P*=0.831 |
| **Language** |  |  |  |  |  |  |
| BNT | r=0.099, *P*=0.437 | r=0.226, *P*=0.072 | r=-0.001, *P*=0.992 | r=-0.019, *P*=0.883 | r=-0.089, *P*=0.482 | r=0.083, *P*=0.514 |
| Category Fluency Test | r=0.104, *P*=0.414 | r=0.227, *P*=0.071 | r=0.197, *P*=0.119 | r=-0.009, *P*=0.943 | r=-0.067, *P*=0.601 | r=0.199, *P*=0.114 |
| **Attention** |  |  |  |  |  |  |
| Log-transformed TMT-A | r=-0.272, *P*=0.030 | r=-0.181, *P*=0.153 | r=-0.038, *P*=0.768 | r=-0.081, *P*=0.527 | r=-0.060, *P*=0.638 | r=-0.375, *P*=0.002* |
| **Executive function** |  |  |  |  |  |  |
| Log-transformed TMT-B | r=-0.164, *P*=0.202 | r=-0.096, *P*=0.459 | r=0.115, *P*=0.372 | r=-0.001, *P*=0.994 | r=0.086, *P*=0.507 | r=-0.238, *P*=0.062 |
| **CSF Biomarkers** |  |  |  |  |  |  |
| Aβ_1-42_ (pg/ml) | r=0.117, *P*=0.357 | r=-0.108, *P*=0.398 | r=-0.124, *P*=0.328 | r=-0.043, *P*=0.734 | r=-0.046, *P*=0.718 | r=-0.025, *P*=0.845 |
| P-tau_181_ (pg/ml) | r=0.012, *P*=0.926 | r=-0.126, *P*=0.322 | r=-0.057, *P*=0.655 | r=-0.140, *P*=0.270 | r=-0.290, *P*=0.020 | r=-0.168, *P*=0.184 |
| T-tau (pg/ml) | r=-0.195, *P*=0.131 | r=-0.207, *P*=0.109 | r=0.122, *P*=0.347 | r=0.141, *P*=0.278 | r=0.107, *P*=0.411 | r=-0.203, *P*=0.117 |
| Abbreviations: sALFF, static amplitude of low-frequency fluctuation; dALFF, dynamic amplitude of low-frequency fluctuation; MFG, middle frontal gyrus; SFG, superior frontal gyrus; MMSE, Mini-Mental State Examination; CDR, Clinical Dementia Rating; WMS-LM, Wechsler Memory Scale Logical Memory; AVLT, Auditory Verbal Learning Test; CDT, Clock Drawing Test; BNT, Boston Naming Test; TMT, Trail-Making Test. * Significantly significant (*P*＜0.01) | | | | | | |

**Supplementary Material 6. The details about MRI acquisition**

The included subjects were from ADNI 2/GO. According to the ADNI 2 Alzheimer’s Disease Neuroimaging Initiative 3T MRI Technical Procedures Manual (<http://adni.loni.usc.edu/wp-content/uploads/2010/05/ADNI2_MRI_Training_Manual_FINAL.pdf>), the 3T fMRI and T1 images in ADNI 2/GO were scanned using the same scanning protocol that initiated in ADNI GO. The details about the scan could be found in <http://adni.loni.usc.edu/wp-content/uploads/2010/05/ADNIGO_MRI_Training_Manual_07292010.pdf>. All resting state functional MRI were scanned on Philips scanners (http://adni.loni.usc.edu/wp-content/themes/freshnews-dev-v2/documents/mri/ADNI_MRI_overview_2.6.18.pdf) .

650 subjects of ADNI-2 that was newly enrolled from approximately 55 sites from the United States and Canada. Approximately 200 EMCI subjects will be followed from the ADNI-GO study from approximately 50 sites. All the MRI scans in ADNI sites followed the standardized acquisition protocols and quality control was made for each image (Jack et al., 2015).

**Supplementary Material 7. The** **comparability of SNAP and predementia AD**

Three groups (NC, SNAP, predementia AD) didn’t differ significantly in the percentage of CN and MCI (Table 1), which ensures the comparability. Moreover, we added the diagnosis as covariates in the imaging comparison of AD and SNAP. The results did not change significantly (eTable 6). Indeed, there might be a discrepancy among the different cognitive stages of SNAP. Due to the limitation of the sample size, we didn’t conduct the subgroup analysis. Future studies with larger sample size and the comparison between the different cognitive stages of SNAP need to be done.

**eTable 6 Brain areas with significant differences of sALFF and dALFF in SNAP and predementia AD (control for age, grey matter volume, and clinical diagnosis)**

| **Neuroimaging Metrics** | **Group** | **Regions** | **Peak MNI** | | | **Cluster Size** | **Peak intensity** |
| --- | --- | --- | --- | --- | --- | --- | --- |
|  |  |  | **x** | **y** | **z** |  |  |
| **sALFF** | **SNAP vs. NC** | Left superior frontal gyrus, Left supplementary motor area, Left paracentral lobule | -12 | -24 | 72 | 120 | -4.61 |
|  |  | Left insula, Left Rolandic operculum, Left superior temporal gyrus | -42 | -9 | 18 | 80 | -4.61 |
|  | **Predementia AD vs. NC** | Right precuneus | 15 | -69 | 48 | 199 | -5.49 |
|  |  | Left superior parietal gyrus, Left inferior parietal gyrus | -24 | -54 | 51 | 73 | -7.62 |
|  |  | Left insula, Left Rolandic operculum, Left superior temporal gyrus | -57 | -9 | 9 | 86 | 4.22 |
|  | **Predementia AD vs. SNAP** | Left middle occpital gyrus, Left superior occpital gyrus | -30 | -69 | 30 | 89 | -5.23 |
|  |  | Left superior parietal gyrus, Left precuneus, Left inferior parietal gyrus | -24 | -54 | 54 | 100 | -5.82 |
| **dALFF variance** | **SNAP vs. NC** | Left superior frontal gyrus, Left supplementary motor area | -18 | 12 | 63 | 83 | -4.48 |
|  |  | Left paracentral lobule | -12 | -24 | 75 | 127 | -5.02 |
|  | **Predementia AD vs. NC** | Left calcarine | -6 | -78 | 12 | 71 | -4.93 |
|  |  | Left middle cingulum, Left post cingulum | -3 | -27 | 33 | 86 | -4.43 |
|  |  | Right middle frontal gyrus | 27 | 33 | 33 | 85 | -4.89 |
|  |  | Right precuneus | 18 | -69 | 48 | 159 | -5.23 |
|  |  | Right superior frontal gyrus, Right supplementary motor area | 15 | -12 | 66 | 81 | -5.06 |
|  |  | Left superior frontal gyrus, Left supplementary motor area, Left paracentral lobule | -18 | 9 | 63 | 88 | -5.27 |
|  | **Predementia AD vs. SNAP** | Left temporal pole: superior temporal gyrus, Left temporal pole: middle temporal gyrus | -36 | 12 | -27 | 75 | -4.78 |
| Statistical significance was set at voxel *P*<0.01, cluster *P*<0.05, controlling for age, grey matter volume, and clinical diagnosis, Gaussian random field (GRF) corrected. **Abbreviations:** MNI, Montreal Neurological Institute; sALFF, static amplitude of low-frequency fluctuation; dALFF, dynamic amplitude of low-frequency fluctuation; NC, normal control; SNAP, suspected non-Alzheimer’s pathophysiology; AD, Alzheimer’s disease. | | | | | | | |

**References**

Jack, C.R., Barnes, J., Bernstein, M.A., Borowski, B.J., Brewer, J., Clegg, S., et al. (2015). Magnetic resonance imaging in Alzheimer's Disease Neuroimaging Initiative 2. *Alzheimer's & dementia : the journal of the Alzheimer's Association* 11(7)**,** 740-756. doi: 10.1016/j.jalz.2015.05.002.

Shaw, L.M., Vanderstichele, H., Knapik-Czajka, M., Clark, C.M., Aisen, P.S., Petersen, R.C., et al. (2009). Cerebrospinal fluid biomarker signature in Alzheimer's disease neuroimaging initiative subjects. *Ann Neurol* 65(4)**,** 403-413. doi: 10.1002/ana.21610.
